# In Vitro Anti-Tubulin Activity on MCF10A Cell Line and In Silico Rigid/Semiflexible-Residues Docking, of Two Lignans from Bursera Fagaroides var. Fagaroides

**DOI:** 10.3390/molecules26206155

**Published:** 2021-10-12

**Authors:** Verónica Rodríguez-López, César Millán-Pacheco, Judith González-Christen, Maricruz Anaya-Ruíz, Omar Aristeo Peña-Morán

**Affiliations:** 1 Facultad de Farmacia, Universidad Autónoma del Estado de Morelos, Cuernavaca 62209, Mexico; veronica_rodriguez@uaem.mx (V.R.-L.); cmp@uaem.mx (C.M.-P.); judith.gonzalez@uaem.mx (J.G.-C.); 2 Laboratorio de Biología Celular, Centro de Investigación Biomédica de Oriente, Instituto Mexicano del Seguro Social, Metepec 74360, Mexico; manaya19@yahoo.com.mx; 3 División de Ciencias de la Salud, Universidad de Quintana Roo, Chetumal 77039, Mexico

**Keywords:** lignans, tubulin, semiflexible docking, MCF10A, podophyllotoxins

## Abstract

Podophyllotoxins are natural lignans with known cytotoxic activity on several cell lines. The structural basis for their actions is mainly by the aryltetralin-lignan skeleton. Authors have proposed a cytotoxic mechanism of podophyllotoxins through the topoisomerase-II inhibition activity; however, several studies have also suggested that podophyllotoxins can inhibit the microtubules polymerization. In this work, the two possible mechanisms of action of two previously isolated compounds from the stem bark of *Bursera fagaroides* var. fagaroides: acetylpodophyllotoxin (1) and 5’-desmethoxydeoxypodophyllotoxin (2), was analyzed. An in vitro anti-tubulin epifluorescence on the MCF10A cell line and enzymatic topoisomerase II assays were performed. The binding affinities of compounds 1 and 2 in the colchicine binding site of tubulin by using rigid- and semiflexible-residues were calculated and compared using in silico docking methods. The two lignans were active by the in vitro anti-tubulin assay but could not inhibit TOP2 activity. In the in silico analysis, the binding modes of compounds into both rigid- and semiflexible-residues of tubulin were predicted, and only for the semiflexible docking method, a linear correlation between the dissociation constant and IC_50_ previously reported was found. Our results suggest that a simple semiflexible-residues modification in docking methods could provide an in vitro correlation when analyzing very structurally similar compounds.

## 1. Introduction

Lignans have been classified according to their chemical structure into eight classes: furan, furofuran, dibenzylbutane, dibenzylbutyrolactone, arylnaphthalene, dibenzocyclooctadiene, dibenzylbutyrolactol, and aryltetralin. Lignans that are metabolized by mammalian gut microflora produce so-called enterolignans [1]. In the Barker publication in 2019, multiple works are reported about the isolation of lignans from plant species, among which are those of the families: Fabaceae, Lauraceae, which is a valuable source of more than 270 lignans and neolignans; in addition to the Mexican plants of the Bursera genus, all of them used in traditional medicine. The strategies for isolation and analysis have been varied, from in vitro and in vivo studies, to in silico studies. Traditionally the health benefits attributed to lignans include lowered risk of heart diseases, menopausal symptoms, osteoporosis, and breast cancer; however, some biological activities have been additionally reported: the anti-inflammatory activity in vitro for the inhibition of 15-lipooxygenase, phospholipases A2, cyclooxygenase 1 and 2 enzyme activities; pharmacokinetics and toxicity (ADMET) profiles to establish if these compounds are lead-like/drug-like compounds [2]; but also further investigations have highlighted the importance of lignan-rich foods and their usefulness to the chronic illness prevention, certain types of cancers and cardiovascular disease, as well as anticarcinogenic, antioxidant, estrogenic, and antiestrogenic activities [3].

Podophyllotoxin (POD), an aryltetralin lignan, was isolated originally from the ethanolic extract of *Podophyllum peltatum*. In 1880 POD caused great expectation for cancer treatment, leading to the characterization of its structure, evaluation of its biological activity, and discovery of its mechanism of action through the inhibition of the tubulin polymerization, a consequent cell arrest in G_2_/M phase and cell death, via necrosis or apoptosis [4,5]. However, despite its great biological potential, its use in clinics was truncated due to its high gastric toxicity and low selectivity [6,7]. Nevertheless, podophyllotoxin has been a pharmaceutically active ingredient in topical solutions and creams to treat the *Condylomata acuminatum* infections [8,9].

**Figure 1:**
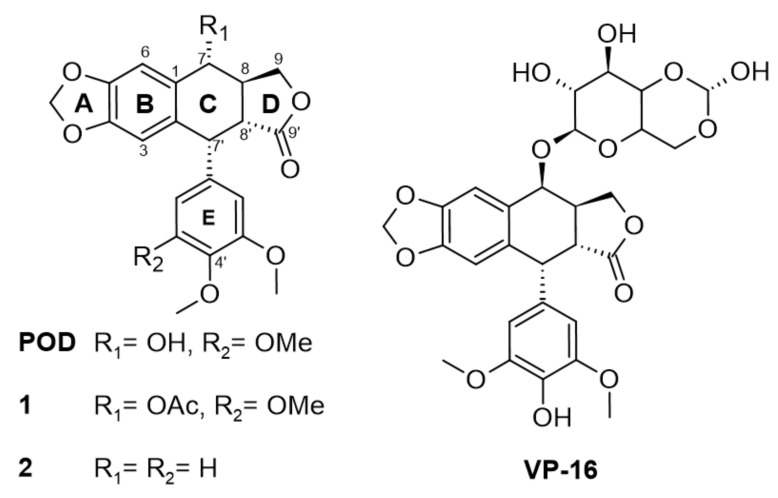
Modifications in structure between podophyllotoxin (POD) and etoposide (VP-16) and compounds 1 and 2.

POD-type lignans are compounds derived from the shikimate pathway, particularly from a phenylpropanoid precursor [10]. Lignans are formed by linking two C_6_C_3_ units and linked by a β-β (8-8′). Aryltetralin-lignans are formed by a system of five rings (A, B, C, D, and E) ranging from the dioxol ring (A), the tetralin ring (B and C) to the γ-lactone ring (D), and an aromatic ring in C-7’ with α configuration (Figure 1). Studies have shown that A and E rings are essential for their activity, and on the other hand, aromatization of ring C leads to loss of action [11].

In Figure 1, the chemical structures of lignan-type compounds used in this work are shown. For example, POD, 1, and 2 share the aryltetralin-type lignan structure, whose differences are found in C-7 and C-4’, for POD a hydroxy (OH-) and a methoxy (OMe-) groups are found, for compound 1, an acetyl group (OAc-) and a methoxy (OMe-), and for compound 2 a hydrogen (H-) substituent in both positions can be found. On the other hand, VP-16 is classified as a semisynthetic epipodophyllotoxin with hydroxyl in C-4’ and an acetylated glucose in the C-7 position.

POD mechanism of action is through a competitive inhibition in the colchicine binding site (named RB3-SLD by Ravelli and collaborators in 2004) [12], and is found among α and β tubulin subunits, and inserted between the strands S8 and S9, T7 loop, and the helices H7 and H8. Colchicine and POD also interact with the T5 loop of the α subunit; this interaction stabilizes the α/β-tubulin heterodimer. For microtubules stabilizations, longitudinal and lateral interactions between the tubulin molecules are required, where the M loops of the tubulin subunits play a central role in these interactions [12].

Etoposide (VP-16) is an epipodophyllotoxin that forms a ternary complex with DNA and topoisomerase II (TOP2) enzyme involved in DNA unwinding. VP-16 (Figure 1) prevents the DNA strands religation causing them to break, resulting in a premitotic block in the late S phase and early phase of G2 [6]. In contrast with POD, VP-16 incorporates demethylation in C-4’ on E ring and an acetylated glucopyranoside in C-7 at C ring [13,14].

Some authors have proposed that the cytotoxic mechanism of POD derivatives is the inhibition of topoisomerase II [15,16,17]. However, several studies have also suggested inhibiting the microtubules polymerization as the cytotoxic mechanism of POD and its analogs [18,19,20].

This study aimed to explore the anti-tubulin effect on the MCF10A cell line (breast cell line), as well as the anti-TOP2 activity of two lignans previously isolated from *Bursera fagaroides* var. fagaroides [21], the compound 1 (acetylpodophyllotoxin) and the compound 2 (5’-desmethoxydeoxypodophyllotoxin) (Figure 1). In addition, the anti-tubulin compound POD, the TOP2-inhibitor anticancer drug VP-16, a lignan-based semisynthetic drug, and taxol (TAX) a microtubules-stabilizer compound [22], were also tested. Additionally, an in silico docking comparison method between rigid and semiflexible analysis in situ was performed with the crystalline structure model of tubulin heterodimer downloaded from Protein Data Bank [23] (code 1SA1). A correlation analysis was done with our previous reported cytotoxic evaluations [24] and the dissociation constant (K_D_) calculated by the affinity energies acquired by the in silico docking methods.

## 2. Results

### 2.1. Microscopy Anti-Tubulin Test

**Figure 2:**
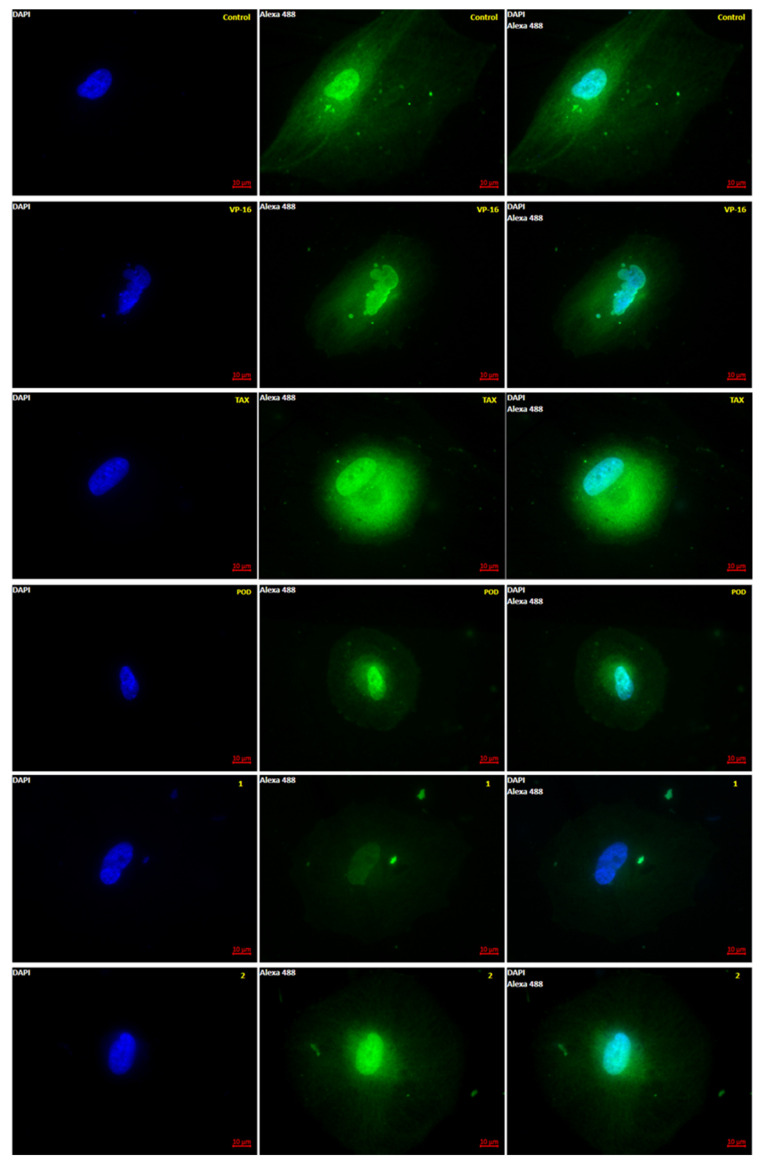
Microtubule polymerization dynamics observed by Epifluorescence. MCF10A cells were stained after 72 h of treatment with tubulin antibody (green) and DAPI (blue). Control was treated with DMSO (0.1%), and VP-16 (0.047 μM), TAX (0.002 μM) [25], POD (0.02 μM), compound 1 (0.318 μM), and compound 2 (0.092 μM). (Magnification 63×-oil).

Anti-tubulin effects of 1, 2, POD, VP-16, and TAX were evaluated by epifluorescence microscopy. Five micrographs were aleatory taken by sample, and each micrograph was measured by a pixel count analysis at the green channel (color of the fluorophore emission from AlexaFluor-488). In Figure 2 a representative micrograph for each treatment after 72 h of incubation is shown.

**Figure 3:**
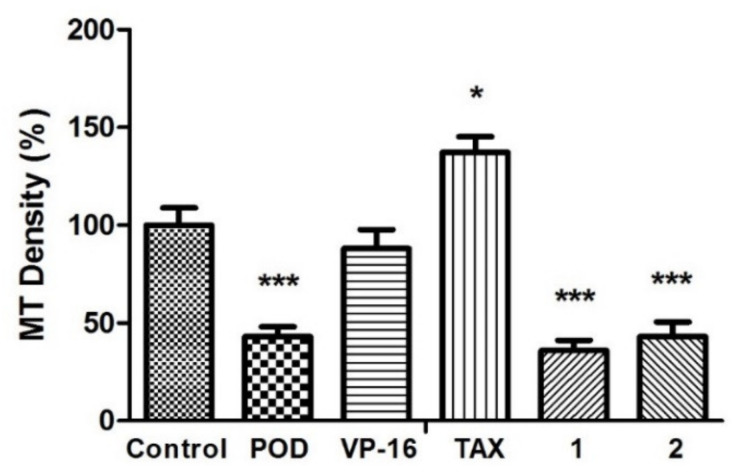
Microtubules densities quantification on MCF10A cells. N = 5 micrographs. Error bars indicate standard deviation (s.d.). Asterisks indicate statistical differences (One-way ANOVA and Dunnett Post-test) * p < 0.05, *** p < 0.001.

It is observed in Figure 2 that the control micrographs showed the conformation of the tubulin cytoskeleton (green), the nucleus (blue), the typical morphology of the MCF10A cells, and their average size. When the cells were treated with the VP-16, it showed microtubules with a low-grade nuclear fragmentation. However, it was also possible to observe cells that have not been modified in the tubulin cytoskeleton. Treated cells with POD presented a decrease in the cellular area and an alteration of the normal morphology. Treatment with TAX showed mostly normal nuclei; however, the signal corresponding to tubulin staining was increased (Figure 3), which could mean that the number of microtubules was also increased. Compounds 1 and 2 showed a decrease in the tubulin signal, but cells with standard size and morphology were also found compared with those of control.

The anti-tubulin activity was evaluated by micrographs analysis, showing that the vehicle did not affect tubulin stability (Figure 3). Each quantitation of relative tubulin content was normalized and compared with the control. When POD was tested, cells were observed with a low cellular area and a loss of normal morphology. The microtubules density percentage (MTD%) obtained was about 43.0% ± 8.6%, statistically different from the control (100% ± 15.4%). Concerning VP-16, there was no statistical difference of MTD% that was about 88.0% ± 17.0%. Treatment with TAX resulted in a high signal of green staining belonging to tubulin stain (137.3% ± 13.6%). A strong tubulin affinity characterizes TAX, and its action mechanism has been reported by promoting tubulin dimerization with stable microtubules formation that is not functional [26]. Compounds 1 and 2 presented a diminished tubulin signal, (35.9% ± 9.0% and 43.0% ± 13.0%, respectively).

To explore the counterpart of the mechanism of action reported by some research groups, the effect of compounds 1, 2, and positive controls (POD and VP-16) on the inhibition of the TOP2 enzyme was determined.

### 2.2. Topoisomerase II Inhibition Assay

**Figure 4:**
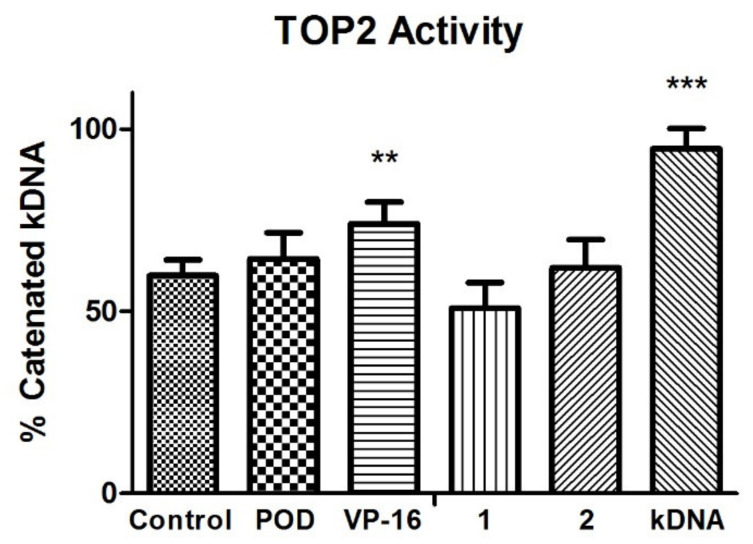
Topoisomerase II (TOP2) inhibition assay. Quantification of remnant kDNA after reaction incubation between TOP2-alfa and compound 1, 2, POD, and VP-16. Densitometric analysis of the agasore gel (n = 2 independent assays). Error bars indicated s.d., and asterisks indicated statistical differences (One-way ANOVA and Dunnett Post-test) ** p < 0.01, *** p < 0.001.

TOP2 mode of action relaxes the supercoiled DNA and decatenates the kinetoplast DNA (kDNA). The VP-16 mechanism of action is over a DNA-TOP2-VP-16 complex formation. Results in the experiment in Figure 4 showed that VP-16 is interfering with the TOP2 action. However, compounds POD, 1, and 2 did not affect TOP2 decatenation properties.

VP-16 showed decreased enzyme activity, unlike POD compounds 1 and 2, which offered a higher amount of decatenated kDNA. Those results suggested that compounds 1 and 2 have no inhibitory activity against TOP2, unlike VP-16. Additionally, the conformations of the compounds were analyzed with an in silico docking study, the results of which are shown below.

### 2.3. Rigid- and Semiflexible-Residues Molecular Docking

To further investigate the effect of inhibiting microtubules formation by compounds 1 and 2, the binding affinities energies between compounds and tubulin (into the colchicine binding site) were calculated. In addition, one bioinformatic analysis was performed for that purpose because no crystalline structure of tubulin from Homo sapiens in complex with POD was found in the PDB database. Therefore, an identity analysis between crystallized tubulin (gene name: TUBB2B) from Bos taurus (UniProt: Q6B856) and Homo sapiens (UniProt: Q9BVA1) was performed and showed 445 identical positions that represent a 100% of identity.

**Figure 5:**
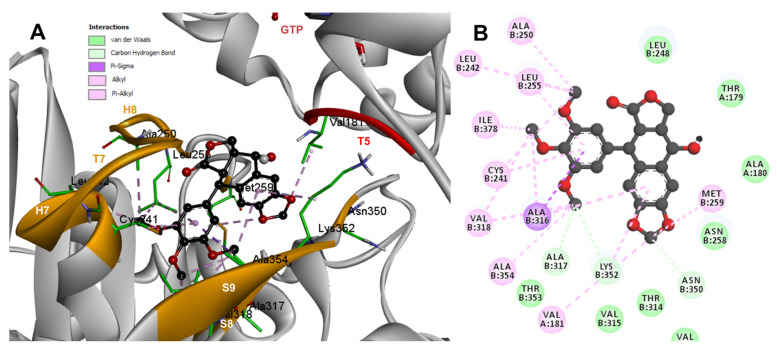
Interactions of POD with the α- and β-tubulin chains and rigid residues. (**A**) A displayed 3D diagram with the suggestive pose obtained by the most populated cluster selected for the conformation visualizations of POD. (**B**) 2D interaction diagram at the RB3-SLD site into tubulin heterodimer and POD. Interaction types between POD and the α- and β-tubulin dimer residues are shown in different colors (residues closer than 4Å).

Based on the computational predictions, POD was inserted between the strands S8 and S9, T7 loop, and the helices H7 and H8. The binding affinity was about −8.6 ± 0.00 Kcal/mol; functional groups interactions with tubulin (rigid residues, Figure 5A) in the colchicine binding site were mainly by hydrophobic interactions between the E ring (trimethoxylated ring) and βAla250, βLeu255, βLeu242, βIle378, βCys241, βVal318, and βAla354, as well as van der Waals interactions were found with βAla317, βThr353. On the other hand, the residues αVal181, βLys352, and βAsn350 interacted with the POD A ring (dioxol) mainly by electrostatics and hydrophobics (Figure 5B).

**Figure 6:**
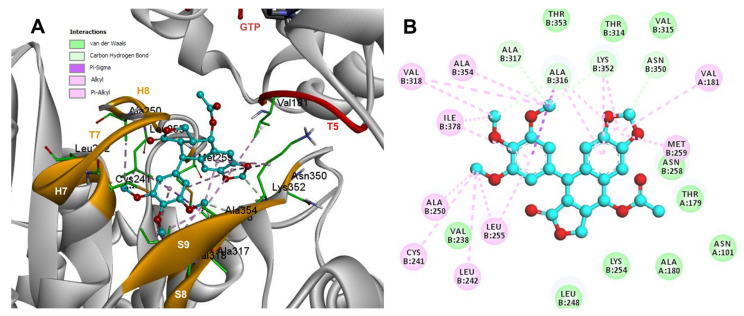
Interactions of 1 with the α- and β-tubulin chains and rigid residues. (**A**) A displayed 3D diagram with the suggestive pose obtained by the most populated cluster selected for the conformation visualizations of 1. (**B**) 2D interaction diagram at the RB3-SLD site into tubulin heterodimer and 1. Interaction types between POD and the α- and β-tubulin dimer residues are shown in different colors (residues closer than 4Å).

**Figure 7:**
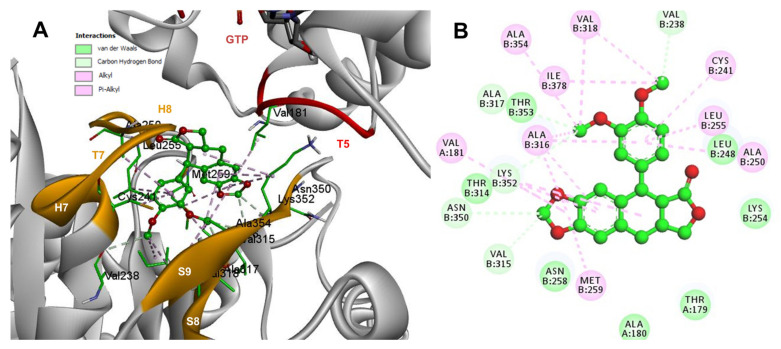
Interactions of 2 with the α- and β-tubulin chains and rigid residues. (**A**) A displayed 3D diagram with the suggestive pose obtained by the most populated cluster selected for the conformation visualizations of 2. (**B**) 2D interaction diagram at the RB3-SLD site into tubulin heterodimer and 2. Interaction types between POD and the α- and β-tubulin dimer residues are shown in different colors (residues closer than 4Å).

Conformations of compounds 1 and 2 were like POD, and both were inserted between the strands S8 and S9, T7 loop, and the helices H7 and H8 (Figure 6A and Figure 7A) of the dimer of α- and β-tubulin computational model. The conformation interactions of compounds 1 and 2 showed tubulin-binding affinities of −6.9 ± 0.03 and −9.4 ± 0.00 Kcal/mol, respectively. Interactions between 1 and residues into the colchicine binding site were mainly by the E and A ring. The residues βAla317, βVal238, βLys352, βAla354, βVal318, βIle378, βAla250, βLeu255, βCys241, βLeu242, and βAla316 were found interacting by hydrophobics with the E ring of compound 1, the residues βLys352, βAsn350, βMet259, and αVal181 also interacted by hydrophobics to the A ring (Figure 6B). For compound 2, residues into the colchicine binding site were mainly by the E (dimethoxylated ring) and A ring. The residues βLue248, βVal238, βLeu255, βAla250, βCys241, βVal318, βIle378, βAla354, βAla316, were found interacting by hydrophobics with the E ring of compound 2, and residues βAla316, βVal181, βMet259, βLys252, βAsn350, βVal315 also interacted by hydrophobics but with the A ring (Figure 7B).

**Figure 8:**
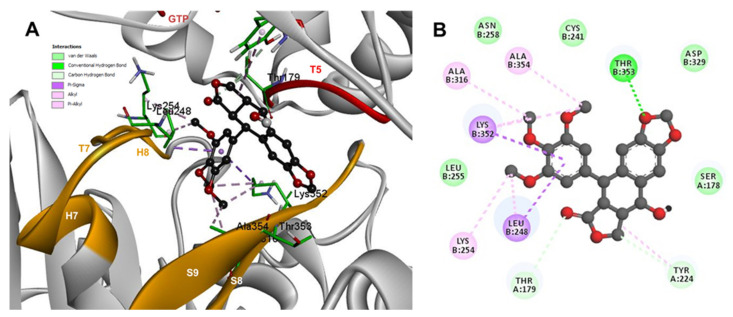
Interactions of POD with α- and β-tubulin chains and semiflexible residues. (**A**) A displayed 3D diagram with the suggestive pose obtained by the most populated cluster selected for the conformation visualizations of POD. (**B**) 2D interaction diagram at the RB3-SLD site into tubulin heterodimer and POD. Interaction types between POD and the α- and β-tubulin dimer residues are shown in different colors (residues closer than 4Å).

The conformations of suggestive poses were reported at the semiflexible residues docking analysis based on the computational predictions. POD was inserted between the strands S9, T7 loop, the helix H8 of the β-tubulin chain, and loop T5 of the α-tubulin chain (Figure 8A). The binding affinity was about −8.6 ± 1.15 Kcal/mol (remained, compared against the rigid docking); the interactions between POD functional groups and tubulin (semiflexible residues, Figure 8B) into the colchicine binding site were mainly by a hydrogen bond (POD as acceptor) formed by βThr353 and the A ring (dioxol), as well as hydrophobic interactions between the E ring (trimethoxylated ring) and βAla354, βAla316, βLys354, βLys352, βLeu248. Furthermore, a hydrophobic interaction was found between αTyr224, and an electrostatic interaction with αThr179, both with the D ring (lactone) (Figure 8B).

**Figure 9:**
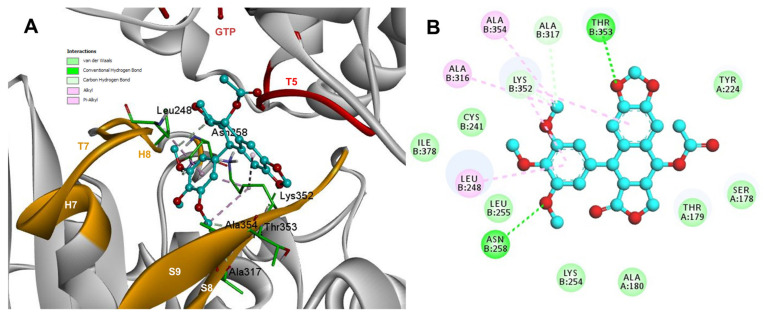
Interactions of 1 with α- and β-tubulin chains and semiflexible residues. (**A**) A displayed 3D diagram with the suggestive pose obtained by the most populated cluster selected for the conformation visualizations of 1. (**B**) 2D interaction diagram at the RB3-SLD site into tubulin heterodimer and 1. Interaction types between POD and the α- and β-tubulin dimer residues are shown in different colors (residues closer than 4Å).

**Figure 10:**
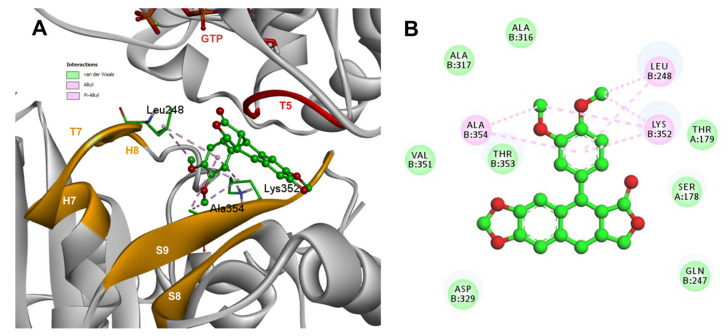
Interactions of 2 with α- and β-tubulin chains and semiflexible residues. (**A**) A displayed 3D diagram with the suggestive pose obtained by the most populated cluster selected for the conformation visualizations of 2. (**B**) 2D interaction diagram at the RB3-SLD site into tubulin heterodimer and 2. Interaction types between POD and the α- and β-tubulin dimer residues are shown in different colors (residues closer than 4Å).

Conformations of compounds 1 and 2 were similar to POD, and both were inserted between the strands S9, T7 loop, the helix H8 of the β-tubulin chain, and loop T5 of the α-tubulin chain of the dimer of α- and β-tubulin computational model (Figure 9A and Figure 10A). The conformation interactions of compounds 1 and 2 showed tubulin-binding affinities of −3.6 ± 1.05 and −10.8 ± 0.81 Kcal/mol, respectively. The E and A ring mainly involved interactions between 1 and semiflexible residues into the colchicine binding site. The residues βAla317, βLys352, βAla354, βAla316, βLeu248 were found interacting by hydrophobics with the E ring of compound 1, as well as a hydrogen bond formed between βAsn258 and the methoxyl group of C-3’/C-5’ (E ring could be rotating). The residue βLys352 was found interacting by a Pi-alkyl type with B ring, and the residue βThr353 formed another hydrogen bond (compound 1 as acceptor) with the A ring of compound 1 (Figure 9B). For compound 2, semiflexible residues into the colchicine binding site were mainly by the E (dimethoxylated ring). The residues βLys352, βLeu248, and βAla354 were found interacting by hydrophobics (Figure 10B).

The calculated affinity energies of POD compounds 1 and 2 in both rigid and semiflexible docking at the colchicine binding site within tubulin, are summarized in Table 1 along with the calculated dissociation constants (K_D_) in molar concentrations. Linearity derived from this comparison is observed in Figure 7A for rigid residues docking and Figure 7B for semiflexible residues analysis. Results revealed a decrease in the affinity energy for compound 1 compared to POD and compound 2 in both computational studies. Those results were correlated with the mean cytotoxic data obtained for the MCF10A cell line in previous studies (IC_50_ POD = 0.019 µM, IC_50_ compound 1 = 0.318 µM, and IC_50_ compound 2 = 0.092 µM) [24]. The data shown in Table 1 suggested a better arrangement of the ligand structures at the RB3-SLD site due to the flexibility in the residues interacting with the POD in its crystallized format. In semiflexible docking, these residues may change their spatial form during the conformational docking search, as would occur in nature. Thus, our study has observed a better interpretation of what could happen between the assayed ligands and the inhibition of the tubulin polymerization. However, the affinity energy of binding has not improved compared to docking with rigid residues.

**Figure 11:**
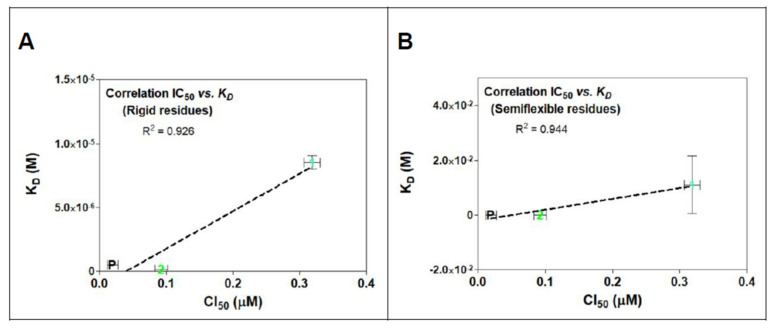
IC_50_ and K_D_ correlations analysis for rigid (**A**) and semiflexible (**B**) residues docking of compounds 1, 2, and POD on tubulin model 1SA1. Linearity is showed in both graphs between K_D_ and cytotoxic assay with MCF10A cells. There is also exposed the determination coefficient (R^2^) from the linear regression analysis.

In Figure 11, a linear relationship between in vitro activity and the proposed mechanism of action for evaluated compounds can be observed. In addition, a simple modified method for semiflexible-residues docking with Autodock vina made it possible to find a (slightly) better linear fit correlation between rigid residues (R^2^ = 0.926, Figure 11A) and semiflexible residues (R^2^ = 0.944, Figure 11B).

## 3. Discussion

MCF10A is a non-tumorigenic human mammary epithelial cell line employed in this study to explore the anti-tubulin effect of two lignans from *Bursera fagaroides* var. fagaroides and the correlations between in silico molecular docking either with rigid or semiflexible residues.

A low amount of quantitated tubulin in the anti-tubulin test probably suggested a destabilization of microtubule polymerized by POD, 1, and 2. Compounds affecting microtubule assembly lead to cell death or an aberrant mitosis exit [27]. Epifluorescence experiment against tubulin showed a changed morphology in cells when treated by 1 and 2, bringing a loss of cytoskeleton area. In addition, treated cells tended to round off. Some cells indicated a condensed and fragmented nucleus, suggesting that a low amount of semi-quantified tubulin could be produced by POD and compounds 1 and 2. Podophyllotoxin-type lignans are known to have cytotoxic activity [28,29,30], and could throw inhibition of the mitotic spindle by the binding with the α- and β-tubulin dimer. However, almost the same structure conformation (epipodophyllotoxins) is also known to be TOP2 inhibitors forming a TOP2-DNA-inhibitor complex [31,32].

Topoisomerase II (TOP2) mode of action relaxes the supercoiled DNA and decatenates the kinetoplast DNA (kDNA). On the other hand, the VP-16 mechanism of action is over a DNA-TOP2-VP-16 complex formation. The statistical analysis at the experiment in Figure 4 showed that VP-16 is interfering with the TOP2 action. However, compounds POD, 1, and 2 did not affect TOP2 decatenation properties.

In the in silico molecular docking analysis, energies calculated for compounds 1 and 2 did not differ considerably from those calculated for POD, suggesting that compounds 1 and 2 could dock to the RB3-SLD site by the same conformation as POD does, and some authors have described the structural basis of the cytotoxic effect of podophyllotoxins through the dioxolan tetralin lactone (A, B, C, and D rings) and the 3’,4’,5’-trimethoxyphenyl fragments [11,33].

The principal interactions of POD-type lignans with β-tubulin chain could be found with the A ring by electrostatics interactions between dioxolane ring oxygens and methylene type carbon with residues of βAsn258, βLys352, and βMet259; and the methylene group between the two oxygen have presented VdW interactions with the βVal315. The B ring (aromatic C-H groups) have been reported mainly VdW-type interactions with βMet259 and βLys352. The C ring (group HO-, for POD) has shown a polar interaction with the αThr179 with the carbonyl group (not reproduced in this docking analysis). The D ring has been found to interact by VdW with the residues of βLeu248, βLys254, and βLeu255, and the trimethoxylated aromatic-type E-ring, have shown VdW interactions mainly with βCys241, βLeu242, βAla316, and βAla317 (Figure 5, Figure 6, Figure 7, Figure 8, Figure 9 and Figure 10).

The most advanced methods for computer-assisted drug design incorporate protein flexibility since these structures have natural movement, and a molecular docking analysis on a rigid protein could be missing some information about the binding between a ligand and a protein [34]. By configuring flexibility to residues in RB3-SLD, it was possible to analyze the changes (searching for better in vitro correlations) between the chemical conformations of each ligand and the binding affinity to tubulin heterodimer.

Comparison analysis between rigid and semiflexible docking allowed to find a linear correlation (Table 1), and by using semiflexible residues configurations, a determination coefficient of 0.944 was calculated, which suggested that although ligands could have some high structural similarities, and simple methods of semiflexible-residues molecular docking configurations could provide a piece of good correlative information with in vitro studies.

With the in silico analysis, the base for the inhibition of tubulin was understood in the colchicine binding site. Similar compounds could have similar mechanisms of action, but the potency of each compound depends on the stability in the binding site into the receptor. In the POD structure, the OH- group in the C ring at C-7 confers high strength to inhibit tubulin polymerization and thereby the capacity to prevent the tubulins heterodimers from passing from a curved structure to a straight one. The hydrogen bond between the C-7 in POD and αThr179 could be fundamental to why affinity decreases when cells are treated by compounds 1 and 2. Our studies suggested an anti-tubulin but not anti-TOP2 bioactivity of acetylpodophyllotoxin (1) and 5’desmethoxydeoxypodophyllotoxin (2) on cell line MCF10A.

However, why compounds 1 and 2 showed differences in their affinity for tubulin or cytotoxic potential remains uncertain since the structural differences are found minimal for compound 2 (lack of a methoxy in the E ring and one hydrogen atom at position C-7). Thus, there may be some other mechanism of action besides tubulin for this class of compounds, other than the inhibition of TOP2.

The mechanism of action proposed to 1, and 2 for the MCF10A cell line is correlated to the works of Antúnez-Mojica, et al., in 2016, for acetylphodophyllotoxin (1), in which a strong interaction with tubulin is reported vs. zebrafish, showing that the E ring is the main group for the interaction with the protein. On the other hand, 5’-desmethoxydeoxypodophyllotoxin (2) has the required structure to exert a favorable interaction to inhibit the mitotic spindle.

## 4. Materials and Methods

POD, VP-16, and TAX were purchased to Sigma-Aldrich (Sigma-Aldrich, Merck KGaA, Saint Louis, MO, USA). Rojas-Sepulveda, 2012 [21] isolated compounds 1 and 2. The MCF10A cell line (ATCC: CRL-10317) was purchased from the American Type Culture Collection (ATCC, Manassas, VA, USA) and cultured in MEGM medium (Lonza group, Basil, Switzerland) in a 5% CO_2_ incubator at 37 °C. Cells were thawed according to the standard methods, and the experiments were performed using freshly thawed cells after three passages.

### 4.1. Microscopy Anti-Tubulin Test

The in vitro anti-tubulin test was performed with MCF10A cells. Briefly, cells (10^4^ cells/mL) were cultured in clean and sterile coverslips (1 cm^2^) inside of 12 wells flat-bottom cell culture plates and incubated in a controlled chamber (5% CO_2_ and 37 °C) for eight hours. Afterward, cells were treated by the half inhibitory concentrations (IC_50_) of POD, VP-16, 1, and 2, each of them calculated in previous work [24], TAX cytotoxic assay concentration (Sigma-Aldrich, Merck KGaA, Saint Louis, MO, USA) were taken by the work of Liebmann, 1996 [26]. Dimethyl sulfoxide (DMSO, Sigma-Aldrich, Merck KGaA, Saint Louis, MO, USA) (0.5%) was used as a control. After 72 h of treatment, cells were fixed with warm formaldehyde (4% in PBS) for 15 min, three times washed with PBS, blocked by Bovine Serum Albumin (BSA) and triton X-100 (2.5% and 0.3% in PBS, respectively) for 60 min. Afterward, the antibody anti-α-tubulin-AlexaFluor 488 (Santa Cruz Biotechnology, Dallas, TX, USA) was added in a BSA solution (1% in PBS) and triton X-100 (0.3% in PBS) and incubated at 4 °C overnight. After incubation, cells were washed three times. Finally, a DAPI (Sigma-Aldrich, Merck KGaA, Saint Louis, MO, USA) solution was added (0.25 μg/mL in PBS) to cells, incubated by 15 min, and washed three times with a PBS solution. Samples were visualized with a Zeiss Observer.Z1 microscope equipped with an Axiocam MRm camera and an Apotome illumination system with a 63x oil immersion objective.

### 4.2. Microtubules Semi-Quantitation

The microtubules semi-quantification was performed using the analyzer images program GIMP (v.2.8, GNOME Foundation, Orinda, CA, USA). The micrographs obtained from the nuclei and microtubule staining assays were processed. For each visualization, cells were selected manually with the selection tool of the software, and the histograms (in the green channel) were obtained for each cell selected. The percentage corresponding to the green pixels was taken from the histogram with the mean value of five analyzed images and plotted as the relative percentage microtubules density (MT density %), according to Formula (1):

MT density (%) = A/B × 100(1)

where A average pixels in the sample (anti-tubulin treated) and B average pixels in control (vehicle-treated).

The MT density (%) for each compound and control were graphed and compared against control with a t-test for unpaired data with a 95% confidence interval.

### 4.3. Topoisomerase II Inhibition Assay

This experiment was performed with a Topogen® kit (TopoGEN, Inc., Buena Vista, CO, USA) and the Topogen’s pure TOP2 enzyme. The assay consisted of a reaction involving the triggering of DNA by the effect of TOP2; VP-16 was used as an inhibition control. Briefly, H_2_O was added to make a final volume of 10 μL, to which was added one μL from buffer A and one μL from buffer B (5X concentrated), one μL from kDNA, 0.5 μL of test compound (4 mg/mL), and 0.5 μL of TOP2 enzyme (10 units/μL). The reaction was incubated in a heating plate for 45 min at 37 °C. Finally, two μL of stop buffer were added. While incubation time was proceeding, a 1% agarose (Amresco, VWR Corporate Headquarters, Radnor, PA, USA) gel was prepared with ethidium bromide (EB, 0.5 μg/mL; Sigma-Aldrich, Merck KGaA, Saint Louis, MO, USA), in which the samples were loaded after the incubation time, DNA was separated by electrophoresis at 100–150 V, with Tris, acetic acid, EDTA buffer (TAE), and photo-documented by an image analyzer Chemi doc MP (Bio-rad^®^). In addition, a linear DNA marker (Xho-I enzyme digestion), a decatenated DNA (DkDNA) marker (TOP2 activity), and a kDNA marker were added.

Quantification of remnant kDNA was obtained by densitometry according to Formula (2):

Catenated kDNA (%) = A/B × 100(2)

where A densitometry analysis in the sample. And B densitometry analysis in control (kDNA).

The Catenated kDNA (%) for each compound and controls (vehicle and kDNA) were graphed and compared with a t-test for unpaired data with a 95% confidence interval.

### 4.4. Flexible- and Rigid-Residues Molecular Docking

**Figure 12:**
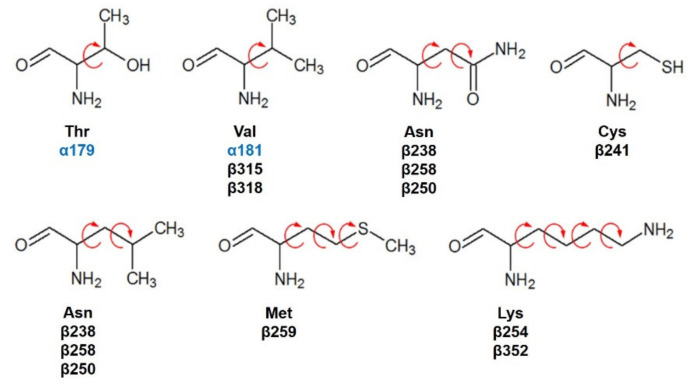
Semiflexible configured residues interacting with POD at 4 Å distance.

Compounds 1 and 2 were docked with the crystallographic heterodimer structure of tubulin from the Protein Data Bank (protein from *Bos taurus* but 100% identity with *Homo sapiens*), PDB code 1SA1, 4.2 Å of the resolution, and cocrystallized with guanosine-5′-diphosphate (GDP), guanosine-5′-triphosphate (GTP), a magnesium ion (Mg^2+^), and POD. POD is found between the polar junction established between the α- and β-tubulin chains (colchicine binding site: RB3-SLD), and the complex has a curved conformation [12]. Amino acids interacting at 4 Å with POD are βGly237, βVal238, βThr239, βThr240, βCys241, βLeu242, βLeu248, βAla250, βAsp251, βLeu252, βLys254, βLeu255, βAsn258, βMet259, βThr314, βVal315, βAla316, βAla317, βVal318, βAsn350, βVal351, βLys352, βThr353, βAla354, and βIle378 belonging to the β-tubulin chain and the amino acids of the α-tubulin chain αThr179, and αVal181. For docking, semiflexible residues were configured using Autodock tools (v.1.5.6, Scripps Research Institute, La Jolla, CA, USA) and manually selected (Figure 12). Docking calculations (semiflexible and rigid configurations) were conducted with Autodock vina (v.1.1.2, Scripps Research Institute, La Jolla, CA, USA), and the grid was positioned at X = 115.9, Y = 90.1, Z = 4.3, with 15 Å by side dimensions, and 0.375 Å separated. Before docking experiments, the protocol was validated by docking the cocrystallized ligand (POD) and obtaining a Root Mean Square Deviation (RMSD) at the rigid of 2.487 Å and semiflexible of 2.480 Å.

Ligands were optimized using an entire energy minimization force field (MMFF94) and docked with dihedral flexibility within the binding site RB3-SLD of the flexible-residues configured protein. For the protein, polar hydrogen atoms were added, and Kollman charges (AMBER) were assigned. Twenty-five independent runs for each ligand were performed. BIOVIA Discovery Studio (v3.5, Dassault Systèmes, Waltham, MA, USA) was used to visualize the final docked conformations for each ligand.

The most populated cluster was examined for rigid and semiflexible docking, and the mean of affinity energies was reported. In addition, the negatives affinity energy of binding was calculated as Kcal/mol, and the K_D_ of each compound was obtained through the Gibbs free energy and equilibrium equation.

### 4.5. Statistical Analysis

The statistical program GraphPad Prism (v.5.00, GraphPad Software, Inc., La Jolla, CA, USA) was employed to determine statistical differences in the experiments. For all statistical tests, significance was established at p < 0.05.

**Figure 13:**
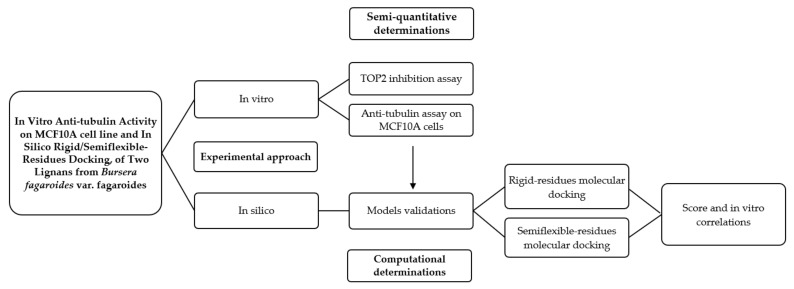
Graphical scheme of the study approach.

In Figure 13, a graphic scheme of the study approach is shown, and the flow of experiments and analysis of this work can be observed, which was carried out with two approaches: the in vitro approach focused on the determination of the possible mechanism of action of two isolated lignans of *Bursera fargaroides* var. fagaroides on a non-cancerous cell line; and the in silico approach, through a molecular docking analysis configured with both rigid and semiflexible residues, the results of which allowed obtaining a better linear correlation with the in vitro cytotoxicity data.

## 5. Conclusions

In conclusion, the treatment with lignans 1 and 2 forms *Bursera fagaroides* var. fagaroides showed less microtubule formation on MCF10A cells (first time reported). Therefore, Aryltetralin lignans are considered with great potential as POD analogs. However, they also share structural similarities with epipodophyllotoxins considered as TOP2 inhibitors. Our results have preliminarily demonstrated that compounds 1 and 2 do not form the TOP2-DNA-inhibitor complex. On the other hand, an in silico docking analysis between the tubulin dimer model configured with semiflexible residues allowed a good correlation between the cytotoxic effect and the ability to decrease the formation of microtubules in the MCF10A line. The favorable interactions within the colchicine binding site in tubulin, added to the microscopic effect observed in the microtubule staining assay, reinforce the hypothesis that podophyllotoxin-type lignans act as potent inhibitors of microtubule dynamics, forming the tubulin-aryltetralin lignan complex.

## Figures and Tables

**Table 1:** Tables display the mean affinity energy of binding (±s.d.) in addition to the mean calculated dissociation constant (K_D_) from each ligand in the lowest energy cluster.

	Rigid Residues		Flexible Residues	
Compound	ΔGb (Kcal/Mol)	K_D_ (M)	ΔGb (Kcal/Mol)	K_D_ (M)
POD	−8.6 ± 0.00	4.96 × 10^−7^ ± 0.00	−8.6 ± 1.15	1.44 × 10^−5^ ± 5.05 × 10^−5^
1	−6.9 ± 0.03	8.53 × 10^−6^ ± 5.08 × 10^−7^	−3.6 ± 1.05	0.011 ± 0.03
2	−9.4 ± 0.00	1.28 × 10^−7^ ± 0.00	−10.8 ± 0.81	3.10 × 10^−8^ ± 5.97 × 10^−8^

## Sample Availability

Samples of the compounds are not available from the authors.
